# New relationships between breast microcalcifications and cancer

**DOI:** 10.1038/sj.bjc.6605873

**Published:** 2010-09-14

**Authors:** R Baker, K D Rogers, N Shepherd, N Stone

**Affiliations:** 1 Biophotonics Research Unit, Gloucestershire Hospitals NHS Foundation Trust, Great Western Road, Gloucester, GL1 3NN,UK; 2 Cranfield Health, Cranfield University (Shrivenham Campus), Shrivenham, Swindon, Wiltshire, SN6 8LA,UK; 3 Department of Histopathology, Gloucestershire department already included Hospital NHS Foundation Trust, Great Western Road, Gloucester, GL1 3NN, UK

**Keywords:** breast cancer, calcifications, carbonate, DCIS, diagnose, FTIR, grade

## Abstract

**Background:**

Breast microcalcifications are key diagnostically significant radiological features for localisation of malignancy. This study explores the hypothesis that breast calcification composition is directly related to the local tissue pathological state.

**Methods:**

A total of 236 human breast calcifications from 110 patients were analysed by mid-Fouries transform infrared (FTIR) spectroscopy from three different pathology types (112 invasive carcinoma (IC), 64 *in-situ* carcinomas and 60 benign). The biochemical composition and the incorporation of carbonate into the hydroxyapatite lattice of the microcalcifications were studied by infrared microspectroscopy. This allowed the spectrally identified composition to be directly correlated with the histopathology grading of the surrounding tissue.

**Results:**

The carbonate content of breast microcalcifications was shown to significantly decrease when progressing from benign to malignant disease. In this study, we report significant correlations (*P*<0.001) between microcalcification chemical composition (carbonate content and protein matrix : mineral ratios) and distinct pathology grades (benign, *in-situ* carcinoma and ICs). Furthermore, a significant correlation (*P*<0.001) was observed between carbonate concentrations and carcinoma *in-situ* sub-grades. Using the two measures of pathology-specific calcification composition (carbonate content and protein matrix : mineral ratios) as the inputs to a two-metric discriminant model sensitivities of 79, 84 and 90% and specificities of 98, 82 and 96% were achieved for benign, ductal carcinoma *in situ* and invasive malignancies, respectively.

**Conclusions:**

We present the first demonstration of a direct link between the chemical nature of microcalcifications and the grade of the pathological breast disease. This suggests that microcalcifications have a significant association with cancer progression, and could be used for future objective analytical classification of breast pathology. A simple two-metric model has been demonstrated, more complex spectral analysis may yeild greater discrimination performance. Furthermore there appears to be a sequential progression of calcification composition.

In the UK incidence rates of breast cancer have increased by 50% over the last 25 years to around 44 000 women and 300 men per annum (DH, 2005). Mammography is used routinely as a method to screen populations for breast cancers, however, only around 10–25% of mammographically suspicious lesions are found to be malignant from tissue biopsy (Evans *et al*, 1999), resulting in unnecessary and costly biopsy procedures. In 2004/05 in the UK 1 749 185 women were screened, of which 85 710 (4.9%) women had further tests and only 13 993 (0.8%) were found to have malignancies present (Evans *et al*, 1999; DH, 2005). This demonstrates that in the UK alone, 71 717 women in 2004/05 had further investigations, including excisional biopsy, with all the associated risks, costs and anxiety involved, when they had no malignancies present.

Breast tissue microcalcifications observed radiologically are frequently employed as a marker for breast pathology type as they can act as a strong differentiator between benign and proliferative (type I and type II, respectively) breast lesions and as an indicator of the presence of an early breast cancer. It is often the presence of type II calcification on mammography that leads to further investigations. However, to date it has not been possible to clearly distinguish between the proliferative lesions on the basis of the presence of type II calcium hydroxyapatite. Around 86% of mammograms have been shown to contain visible microcalcifications in women aged 76–79 years (DH, 2005). Survival of patients with mammographic microcalcifications is significantly shorter than those without (Ketcham and Muffat, 1990; Holme *et al*, 1993) and a significantly larger number of lymph nodes are involved in patients with tumours showing microcalcifications than those without (Holme *et al*, 1993).

Furthermore, as the introduction of mammographic screening in the UK, the incidence of ductal carcinoma *in situ* (DCIS) has increased from 2 to 20% of all cancers presenting (Office for National Statistics: registrations, 2000), with 80% of DCIS being detected because of the microcalcifications in the mammogram. It has been shown that 50% of patients with microscopic foci of DCIS develop invasive carcinoma (IC) (Page *et al*, 1982), and that progression to invasion is related to grade. The most advanced form of DCIS: comedo disease, progresses into IC both more often and more rapidly than low grade DCIS (Office for National Statistics: registrations, 2000). However, there is no clear consensus among pathologists (over six different classification systems)—there remain major discrepancies in DCIS grading.

Both mammography and tissue biopsy procedures can detect changes in tissue morphology, but generally do not identify the underlying biochemical changes that distinguish benign from malignant tissue.

## 

### Microcalcifications

Microcalcifications are divided into type I (calcium oxalate) and type II (carbonated calcium hydroxyapatite—CHAP). Type I are mainly associated with benign breast disease. However, type II are the most frequently observed calcifications (Haka *et al*, 2002), and can be found in both benign and malignant proliferative lesions, although they are mostly found to be associated with malignancy. Apatite calcification in other biological tissues is generally composed of calcium-deficient, carbonated hydroxyapatite (Pleshko *et al*, 1991) with most of the carbonate ions substituting at the phosphate site up to 5–6 wt% phosphate. The carbonate substitution within such bioapatites has the effect of significantly increasing the minerals’ chemical reactivity (Nelson, 1981).

Differences have previously been found in the biochemical composition of type II microcalcifications occurring in benign and malignant tissue (Haka *et al*, 2002). It was found from Raman spectroscopic analysis of breast microcalcifications that carbonate content is greater in type II (CHAP) calcifications from benign than malignant breast tissue. However, beyond this, there remains very little chemical information concerning these microcalcifications. It would, therefore, be of great value to investigate further the biochemical nature of these type II microcalcifications and to determine whether particular features are characteristic of different pathology types and sub-grades.

The study outlined here aims to determine the carbonate content and the amount of CHAP mineral in comparison to tissue matrix, in type II microcalcifications, in three distinct pathology types (benign, *in-situ* and invasive) and also between invasive cancer grades (grades 1, 2 and 3) and stages of DCIS (low, intermediate and high grade (HG)). The association of calcification chemistry with increasing histological grade of tissue will also be examined.

The combination of relative carbonate content with other parameters of a biochemical nature would be of great value in diagnosis, allowing the possibility of differentiation of tissue pathology on the basis of the direct analysis of CHAP spectra. The combination of spectral acquisition with microscopy, would allow direct comparison of biochemical information with histology, possibly leading to a more accurate diagnosis. Furthermore, there are advancing forms of Raman spectroscopy that would allow this information to be obtained non-invasively using low power laser light (Haka *et al*, 2002; Crow *et al*, 2005). Measurement of calcification spectra from a depth within mammalian and human breast tissues has already been shown to be possible (Baker *et al*, 2007; Stone *et al*, 2007a, 2007b). Recent advances have demonstrated that composition could be elucidated through 27 mm of porcine tissue (Stone and Matousek, 2008), with further developments expected (Stone and Matousek, 2008) to make this possible for breast thicknesses of 3–5 cm.

## Methods

### Specimens

Breast tissue specimens were collected from 110 patients of different pathology types (21 benign, 31 *in-situ* carcinoma (low–high grade) and 56 invasive-grades 1–3). Out of these 110 specimens, 106 (21 benign, 30 *in-situ* and 55 invasive cancer) were analysed by FTIR spectroscopy, as no calcifications could be found in the others. Altogether, 236 calcifications were analysed (60 benign, 64 *in-situ* and 112 invasive). The specimens were selected from examination of radiographs from archives of patients who had undergone biopsy for mammographically suspicious breast lesions at Gloucestershire Royal Hospital between 1996 and 2007 (ethical approval Gloucestershire Local Research Ehics Committee). Samples that were known, from histopathology reports, to contain calcifications were chosen. The paraffin-set biopsy blocks were then retrieved from storage, microtomed to 7-*μ*m thickness and mounted onto calcium fluoride (CaF_2_) slides for FTIR analysis. The Nottingham grading system was used (Ellis *et al*, 2005) to define the pathologies of the tissues surrounding the calcifications in this study. This is the system used for routine breast pathology in Gloucestershire Hospitals, UK.

Invasive samples included invasive ductal and invasive lobular carcinoma (ILC) of varying grades between 1 (least aggressive) and 3 (most aggressive). *In-situ* samples ranged from ductal carcinoma to lobular carcinoma of high, intermediate and low grades. Benign samples included atypical ductal hyperplasia, usual type hyperplasia, fibroadenoma, fibrocystic change, papillary lesions, periductal elastosis, duct ectasia and sclerosing adenosis.

It should be noted that two benign calcifications came from samples also exhibiting DCIS (intermediate grade); four DCIS calcifications were from the tissue containing both low and HG; the five lobular carcinomas *in situ* were found in mixed samples with DCIS; one sample contained both ILC and invasive ductal carcinoma (IDC). Otherwise samples were classified as pathologically homogeneous.

### Experimental

#### FTIR instrumentation

Samples were measured by Fourier transform mid-infrared (mid-FTIR) microspectroscopy using a Perkin Elmer Spotlight 300 system (Seer Green, Buckinghamshire, UK), incorporating a 16 × 1 element (400 × 25 *μ*m) mercury–cadmium–telluride array detector and a single point, 100 × 100 *μ*m mercury–cadmium–telluride detector. Both detectors were liquid-nitrogen cooled. The detectors are sensitive over the spectral range from 720 to 4000 cm^−1^, which is equivalent to wavelengths of around 14 and 2.5 *μ*m, respectively. Visible images were collected under white light LED illumination by a Charge Coupled Device camera, which allowed selection of regions for imaging.

#### Mapping/point measurements of paraffin-embedded specimens

**Figure 1:**
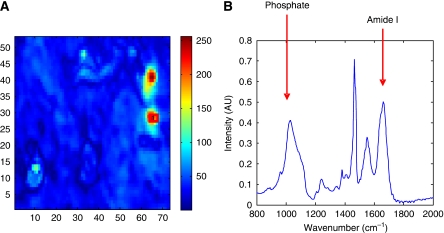
(**A**) An example of how mean regions were selected, showing a false colour image of PC score 3 of an invasive cancer sample with selected regions indicated by red boxes. (**B**) Average spectra of those collected from area within red box in (**A**). Absorbance peaks corresponding to vibrational modes from the phosphate (around 1020 cm^−1^) and amide I (1650 cm^−1^) are indicated by arrows.

Tissue sections containing calcifications, and the locally surrounding tissue were imaged using a pixel size of 6.25 *μ*m and a 4 cm^−1^ spectral resolution with 64 scans per pixel. One or two image maps were taken per sample, containing a total of 236 calcifications and averaging around 7000 spectra per map. A mean spectrum was then taken of the areas of calcification from the spectral image map, and used for calculation of amide: phosphate ratios and for partial least square calculation of carbonate content (see Figure 1).

#### Point measurements of CHAP standards for partial least square model construction

**Figure 2:**
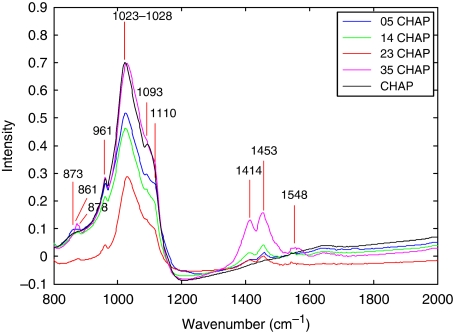
Mean spectrum from each CHAP standard ranging from 0 to 3.5% carbonate substitution.

The CHAP standards were obtained (Clarkson Chromatography Products, South Williamsport, PA, USA) in powder form, sprinkled finely onto CaF_2_ slides and measured in transmission mode. Point spectra of CHAP standards containing pure hydroxyapatite and CHAP of 0.5, 1.4, 2.3 and 3.5% carbonate incorporation, were collected on the FTIR system. Spectra were taken from 10 areas on each CHAP/HAP sample. Each spectrum was acquired with 64 scans and a 4 cm^−1^ resolution, with a spectral range of 720–4000 cm^−1^. Background scans were obtained from a blank section of the CaF_2_ slide and a ratio was obtained against the sample spectrum each time. The average spectrum from each standard is shown in Figure 2. As the carbonate substitution increased (from 0 to 3.5%), the spectra showed an increasing intensity (compared with that of the baseline) of all carbonate peaks, most easily seen by looking at the doublets appearing at around 1410 and 1450 cm^−1^.

**Figure 3:**
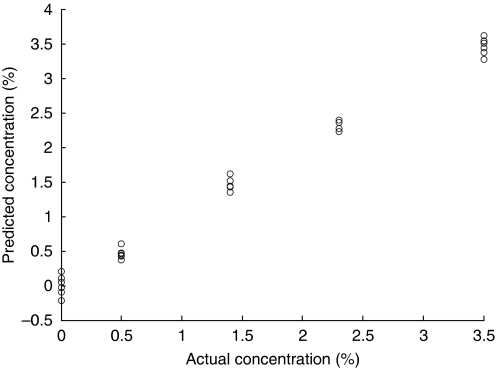
Partial least square model showing actual against predicted % carbonate content in the 49 measured CHAP spectra.

From the measurements of these CHAP standards, a partial least squares prediction model was constructed, which enabled calculation of the amount of carbonate present in unknown samples by analysis of their spectral patterns. Initially, the spectra were truncated to 750–2000 cm^−1^ and converted to second derivative spectra, which have been shown to be optimal for many FTIR spectral analysis models (Paschalis *et al*, 1996; Gao *et al*, 1999; Beleites *et al*, 2005; Das *et al*, 2007). The partial least square model was constructed with four latent variables from the 49 CHAP spectra, which described over 93% of the data, without including other factors that may result in over-fitting. A standard error of the mean was calculated as ±0.17% (CO_3_^2−^ substitution). Figure 3 shows the predictive value of the model when tested using leave-one-out cross-validation.

## Results

**Figure 4:**
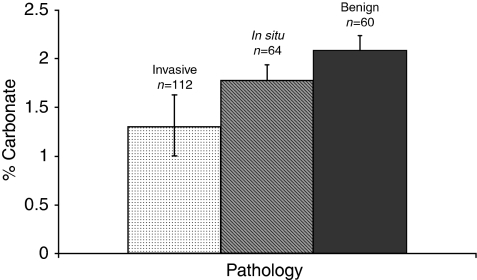
Mean carbonate content in calcifications from invasive, *in-situ* and benign breast tissue (mean values are 1.41 for invasive; 1.83 for *in-situ* and 2.08 for benign). Error bars show the standard deviation of the values measured. *P*-values calculated from Student's *t*-tests of the data were 5 × 10^−40^ (invasive *vs* benign), 1 × 10^−21^ (invasive *vs in-situ*) and 3 × 10^−20^ (*in-situ vs* benign) (total number of calcifications=236).

Figure 4 displays the measured increase in the carbonate composition of the breast calcifications from invasive through to benign breast tissue. When the groups are combined into the three key groups of invasive, *in situ* and benign disease mean carbonate values were found to be 1.41, 1.83 and 2.08%, respectively. These differences were greater than 99.9% signficiant with *P*-values calculated from Student's *t*-tests of the data of 5 × 10^−40^ (invasive *vs* benign), 1 × 10^−21^ (invasive *vs in-situ*) and 3 × 10^−20^ (*in-situ vs* benign).

**Figure 5:**
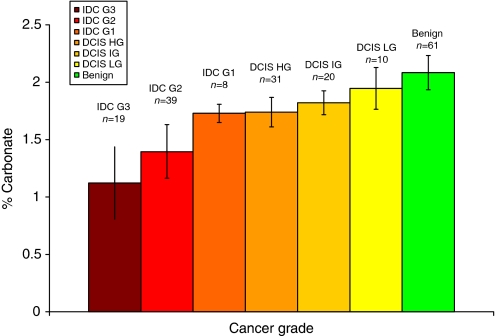
Mean carbonate content in calcifications from all invasive, *in-situ* and benign breast tissue groups studied. Error bars show the standard deviation of the values measured.

The carbonate content was also compared for grades of invasive cancer (invasive ductal carcinoma and ILC) ranging from grade 1 (least invasive) to grade 3 (most invasive) and grades of ductal carcinoma *in-situ* (DCIS) (see Figure 5). A decrease in carbonate content was seen from low- to high-grade DCIS and from grades 1–3 of invasive cancer. Population testing showed that the carbonate values associated with each invasive cancer grade were significantly different (*P*<0.001). The carbonate content was not significantly different between low and intermediate grade DCIS calcifications, although it was (*P*<0.001) significant between low and HGs and intermediate and high-grades of DCIS.

**Figure 6:**
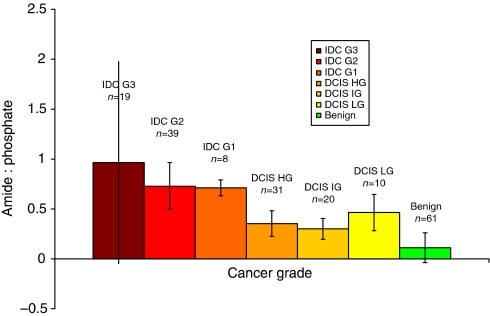
Mean amide : phosphate ratios for calcifications from all pathology groups studied. Mean values for the three groups of invasive, *in-situ* and benign breast tissue calcifications were 0.8278, 0.3717 and 0.1114, respectively, *P*-values calculated from Student's *t*-tests of the data were 2.3 × 10^−16^ (invasive *vs* benign), 1.5 × 10^−8^ (invasive *vs in-situ*) and 2.6 × 10^−15^ (*in-situ vs* benign) (total number of samples=236).

An increase in the matrix : mineral (amide : phosphate) ratio was seen in calcifications from increasingly malignant tissue. Significant differences were observed between benign, *in-situ* and invasive tissue types, when grouped together, see Figure 6 (*P*<0.001). No significant differences in amide : phosphate ratio was observed between invasive cancer grades or between DCIS grades (*P*>0.001).

**Figure 7:**
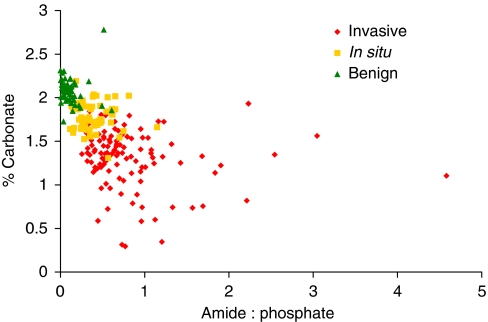
Scatter plot showing separation of calcifications from invasive (*n*=112), *in-situ* (*n*=64) and benign (*n*=60) pathology groups based on carbonate content and amide to phosphate ratios.

By using the two parameters outlined above, each calcification measured could be marked on a scatter plot by the relative level of carbonate concentration and amide : phosphate ratio. This is shown in Figure 7. The plot demonstrates the clustering of calcifications by tissue pathology. It can be seen that malignant samples cover a larger distribution of values than the benign specimens measured. Furthermore the two metrics could be used as inputs to a linear discriminant model. Tables 1 and 2 show the performance of this model for discrimination between the three key pathologies using the calcification spectra.

An assessment was made of the variation between calcification composition from breast disease of different origins. Supplementary Figure S1 demonstrates the difference in these spectral parameters between IDC and ILC). Only six ILC specimens were obtained (one containing an additionally diagnosed IDC). All five containing only ILC appeared to have spectral parameters that lay within the same range as the IDC calcifications, although all had amide : phosphate ratios of less than 1%, and carbonate contents greater than 1%.

The *in-situ* carcinoma specimens all consisted of DCIS, with five containing both LCIS and DCIS. The spectral measurement of carbonate and amide : phosphate ratios from calcifications for these five samples (Supplementary Figure S2) appeared to lie within the normal range expected for calcifications from DCIS tissue.

A further examination was made between potentially associated grades of disease. The carbonate content between high-grade DCIS and invasive cancer G1 was not significantly different (*P*=0.54), the amide to phosphate ratios were also not statistically significant, although it came close (*P*=0.0014). A *P*-value of 0.007 was achieved when testing the difference between carbonate content between low-grade DCIS and ICs G1, whereas the statistical difference between moderate-grade DCIS and IC G2 and high-grade DCIS and IC G3 were once again highly significant with *P*-values <10^−10^.

## Discussion

Significant differences in carbonate content between the microcalcifications of benign, *in-situ* and invasive breast tissues were observed. Furthermore, significant differences were also demonstrated between invasive cancer grades (G1-3) and for ductal carcinoma *in-situ* (DCIS) between high- (HG) and low-grade and HG and intermediate grade. A key finding was that a decrease in carbonate content followed a continuum from benign to increasingly malignant tissue.

Differences were greater between the invasive cancer grades than between the DCIS grades, indicating that as the tissue increases in malignancy, the carbonate content within corresponding calcifications decreases at a greater rate. There was less of a decrease in carbonate substitution between increasing DCIS grades and DCIS HG and IDC G1 were similar in their carbonate content, suggesting the calcification in high-grade DCIS is in a chemical state similar to that found within early invasive disease. This indicates that at least in terms of calcification formation and tissue physiology these groups are very similar. Therefore, a second key finding was that the data in this study supports a continuous pathology model from *in-situ* and invasive tissues. It must be noted that molecular biological methods have demonstrated that there are similarities in cellular molecular expression between the following pairs of groups of low-grade DCIS and IDC G1 and high-grade DCIS and IDC G3 (Abdel-Fatah *et al*, 2008).

The increased carbonate in benign lesion calcifications may be related to the reactivity of CHAP. Previous studies have shown that an increase in carbonate within the CHAP lattice directly increases its reactivity and solubility. The increased concentration of carbonate results in a less stable structure (Porter *et al*, 2005) and upregulated interaction with other ions (Legeros and Legeros, 1993; Miyaji *et al*, 2005), which may have an inhibitory effect on its biological activities associated with cancer progression, such as preventing activation of certain enzymes. For example, it has been suggested that certain matrix metalloproteases are stimulated by the presence of CHAP (McCarthy *et al*, 1992; Reuben *et al*, 2002; Sun *et al*, 2003; Zeng *et al*, 2003).

Many spectra collected from microcalcifications possessed a contribution from characteristic soft tissue vibrational modes of DNA, phospholipids and protein. In particular there was a noticeable difference in the intensity of the amide I and II peaks with different pathology type and grade. Perhaps, it is surprising that the appearance of a tissue ‘matrix’ signal was also seen from within the centre of many calcifications. Data were collected with an image pixel size of 6.25 *μ*m (and wavelengths of between 5 and 14 *μ*m) and a section thickness of 7 *μ*m. These dimensions are significantly smaller than any of the examined calcifications, therefore, the presence of tissue matrix within the calcification would be extremely unlikely to be due to measurement of the surrounding tissue area.

The increase in the matrix : mineral ratio in calcifications in increasingly malignant tissue appears to be due to an increase in the amide/protein content, rather than a reduction in the phosphate content. Invasive tissue generally has an increased cellular activity and higher protein content (Frank and McCreery, 1995). Our study also supports the findings of Haka (Haka *et al*, 2002), in which an increase in protein was observed with Raman spectroscopy in calcifications in invasive compared with benign tissue. However, it may also be possible that the total mineral content is greater in calcifications of benign lesions. Benign calcifications are typically described as being more crystalline, although this also includes examination of type I (calcium oxalate) calcifications (Tse *et al*, 2007). The fact that the nature of calcifications (carbonate content and matrix : mineral ratios) in DCIS pathology, appears to lie consistently between benign and invasive types, indicates that benign tissue calcifications (consisting of fibroadenoma, ductal hyperplasia and fibrocystic change) are likely to lead to a DCIS, which in turn will result in invasive disease. Although this appears intuitive it is not a universally accepted concept. Further insight would be gained by following up patient disease progression to support the information found when measuring the archived breast calcification from needle biopsy.

This study has demonstrated that benign, *in-situ* and invasive pathologies can be well discriminated using FTIR spectral measurements to calculate both carbonate content and matrix : mineral ratios of calcifications (Figure 7, Tables 1 and 2). This produces important implications for diagnosis of disease by calcification measurement using FTIR spectroscopy or other techniques, such as deep Raman spectroscopy (Stone *et al*, 2007b, Stone and Matousek, 2008). The determination of these physio-chemical parameters from invasive tissue calcifications appeared to be spread over a much larger range and only minimally overlapped with the *in-situ* type. There was also a slight overlap of benign with *in-situ*, but benign and invasive types were fully separated, which is an important observation in terms of diagnosis. This aspect has been further investigated by the use of linear discriminant analysis prediction models (two-metric model performance shown in Tables 1 and 2) that can provide a method of using this data for accurate diagnostic prediction. Further exploration of multivariate approaches for more accurate discrimination is beyond the scope of this paper.

## Figures and Tables

**Table 1:** Confusion matrix of performance of model for each calcification *vs* histopathology of the surrounding tissue

	**Two-metric prediction**		
	**Benign**	**DCIS**	**Invasive cancers**
*Histopathology*			
Benign	88	24	0
DCIS	3	54	7
Invasive cancers	0	6	53

Abbreviation: DCIS=ductal carcinoma *in situ.*

**Table 2:** Performance of two-metric disciminant model

	**Benign**	**DCIS**	**Invasive cancers**
Sensitivity per %	79	84	90
Specificity per %	98	82	96

Abbreviation: DCIS=ductal carcinoma *in situ.*
