# Supplementary material for: New relationships between breast microcalcifications and cancer
Source: Br J Cancer. 2010 Sep 14;103(7):1034–9. doi: 10.1038/sj.bjc.6605873 (PMC2965876; doi:10.1038/sj.bjc.6605873)
Supplement: Supplementary Figures S1–S2 [file 6605873x1.doc]

**Supplementary Figures**


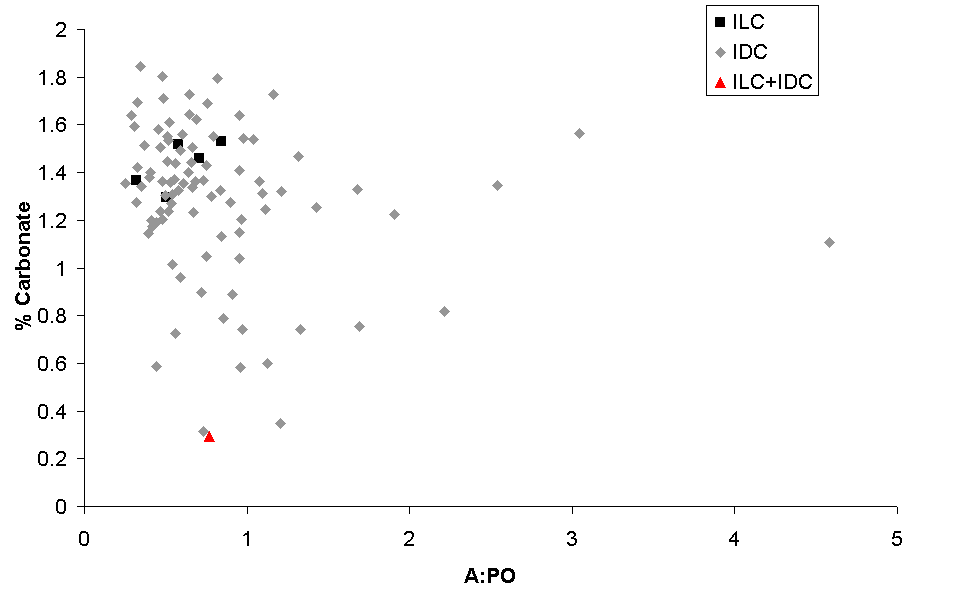


Figure S1. Scatter plot showing a comparison of carbonate content and amide: phosphate ratios in calcifications from invasive ductal carcinoma (IDC) (n=106) and invasive lobular carcinoma (ILC) (n=5) (and one sample containing both IDC and ILC).


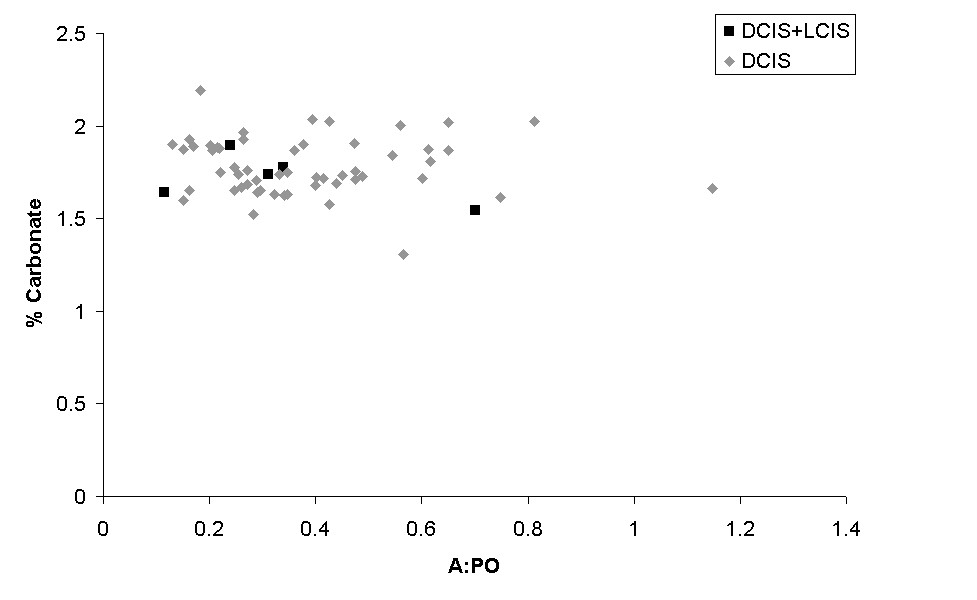


Figure S2. Scatter plot showing a comparison of carbonate content and amide: phosphate ratios in calcifications from ductal carcinoma in-situ (DCIS) (n=59) and DCIS with lobular carcinoma in-situ (LCIS) (n=5).
